# Restoring black triangle with bioclear matrix versus conventional celluloid matrix method: a randomized clinical trial

**DOI:** 10.1186/s12903-023-03102-y

**Published:** 2023-06-17

**Authors:** Aya Omar Tawfik Hussien, Shereen Hafez Ibrahim, Mona El Saied Essa, Randa Mohamed Hafez

**Affiliations:** 1 Department of Conservative Dentistry, Faculty of Dentistry, Cairo University, 11 EL-Saraya St. Manial, Cairo, 11553 Egypt; 2 Faculty of Dentistry, Beni-Suef University, Beni Suef, Egypt

**Keywords:** Black triangle, Injection molding technique, Restorative regenerative papilla, Bioclear matrix, Celluloid matrix

## Abstract

**Background:**

Open gingival embrasures form complex aesthetic and functional problems. This clinical trial assessed the bioclear matrix using injection molding technique against conventional celluloid matrix technique in management of black triangle.

**Methods:**

A total of 26 participants were randomly divided into two groups (13 participants each) according to the technique used. In group (A) celluloid conventional matrix method was used, while in group (B) bioclear matrix with injection molding technique was used. The different outcomes (Esthetic evaluation, marginal integrity and patient satisfaction) were evaluated following the FDI criteria by two blinded examiners. The evaluation was done at (T0) (immediate after restoration); (T6) after 6 months; and (T12) after 12 months. Statistical analysis was done as categorical and ordinal data were presented as frequency and percentage values. Categorical data were compared using fisher’s exact test. Intergroup comparisons for ordinal data were analyzed utilizing the Mann–Whitney U test, while intragroup comparisons were analyzed using Friedman’s test followed by the Nemenyi post hoc test. The significance level was set at *p* ≤ 0.05 within all tests.

**Results:**

Regarding radiographic marginal integrity and marginal adaptation, the bioclear matrix group revealed superior results when compared to celluloid matrix group with a significant difference between both groups at all intervals (*p* < 0.05); however no significant difference was detected at different intervals. While for proximal anatomical form and esthetic anatomical form, as well as phonetics and food impaction, all cases in both groups were successful with no statistical significant difference between groups. For the periodontal response, there was no significant difference between groups. However, there was a significant difference between scores measured at different intervals, with T0 being significantly different from other intervals (*p* < 0.001). Marginal staining revealed that there was no significant difference between groups. While, a significant difference between scores measured at different intervals.

**Conclusions:**

The restorative management of the black triangle with both protocols was able to deliver superior aesthetic and good marginal adaptation; suitable biological properties; with adequate survival time. Both techniques were almost equally successful, however they are depending on the operator skills.

**Trial registration:**

The clinical trial was registered in the (www.clinicaltrials.gov/) database in 23/07/2020; with the unique identification number NCT04482790.

## Background

In recent years, there has been an increase in the demand for cosmetic dentistry to improve a person's appearance. The demand for aesthetics has increased the periodontists' ability to overcome aesthetic problems in patients [1]. A recent study of patients’ attitude found that there is patient's dissatisfaction with the black triangles in which these imperfections rank quite high among aesthetic defects. Their level is the third followed carious lesions and dark crown margins [2]. Open gingival embrasures or black triangles form complex aesthetic and functional problems. There are several drawbacks such as they are remarkably non-aesthetic and adversely affects the smile, facilitate retention of food debris which can affect the health of the periodontium negatively [3].

Numerous non-surgical and surgical treatments for soft tissue abnormalities and interproximal spaces have been proposed. Regarding nonsurgical treatments, they include repeated papillae curettage, restorative intervention, orthodontic treatment, and prosthetic treatment [2]. As a guide for the formation of an interdental papilla, a resin composite can be inserted near the gingival sulcus [4]. In terms of papillary regeneration, restorative modalities are used to guide the shape of the interdental papilla, for instance, composite resin laminates and crowns can be extended into the gingival sulcus. The most popular matrix technique for proximo-incisal defects is the use of a contoured clear mylar strip. The main benefits of using the mylar strip are undoubtedly its simplicity and speed [5].

The adequate proximal contour and contact of the restoration are one of the challenges experienced when performing direct restoration involving the proximal walls. As a result, several studies have been conducted to investigate techniques and materials able to re-establish proper proximal contact tightness. In this regard, the technique used is known to have an impact on the proximal quality. A proper proximal contact tightness and contour are critical in balancing the dental element as well as periodontal health [6].

Despite the fact that the ideal balance of pink and white was not achieved, the bioclear method is a unique alternative solution for finishing orthodontic cases. The method focuses on white rather than pink, as well as the macro-aesthetics of the smile since most patients' aesthetic desires are met while their dentition is preserved. Moreover, the use of modern materials to restore lost gingival tissue and improve aesthetics, such as gingival-colored resin composite, can be a simple and cost-effective way to manage patients with generalized aggressive periodontitis [7].

Since our first concern was to focus on the most conservative solutions in dealing with the missed interdental papilla and based on the literature found as a result of the search process, a limited number of them were reached, and unfortunately, they were low evidence only limited to case reports [7–10]. Therefore, it was useful to assess the bioclear matrix and injection molding technique against conventional celluloid matrix technique in management of black triangle. The null hypothesis was that there will be no significant difference in using celluloid conventional matrix and bioclear matrix for black triangle restoration after 0,6,12 months regarding the three outcomes (esthetic evaluation, marginal integrity and patient satisfaction).

## Methods

The tested Armamentarium used in this study are: Celluloid matrix (for the control group) which is transparent strip (TOR VM, Russia) with 8 mm width, 95 mm length, and 0.05 mm thickness and Bioclear matrix black triangle kit (for the intervention group) consisting of Black Triangle Matrix Series (Bioclear matrix. USA) of color-coded matrices that close space up to 2.5 mm, true contact saw and sanders, black triangle gauge, and bioclear dual-color disclosing solution for both groups.

### Study setting

This randomized controlled clinical study was held in the Faculty of Dentistry, Cairo University, Egypt. The protocol of the study was revised and approved on the 7^th^ of August 2019 by the committee of the research plan and evidence-based in the Conservative Dentistry Department, Faculty of Dentistry, Cairo University, Egypt. The number of the involved participants in this study was calculated, revised, and approved by Medical Biostatistics Unit, Faculty of Dentistry, Cairo University. The clinical procedures and logistics of this clinical trial were approved by the Research Ethics Committee of the Faculty of Dentistry, Cairo University with approval number 3592019 and in accordance with the Declaration of Helsinki and its later modifications. Then, the protocol of the clinical trial was registered in the (www.clinicaltrials.gov/) database in 23/07/2020; with the unique identification number NCT04482790.

### Study design

**Fig. 1:**
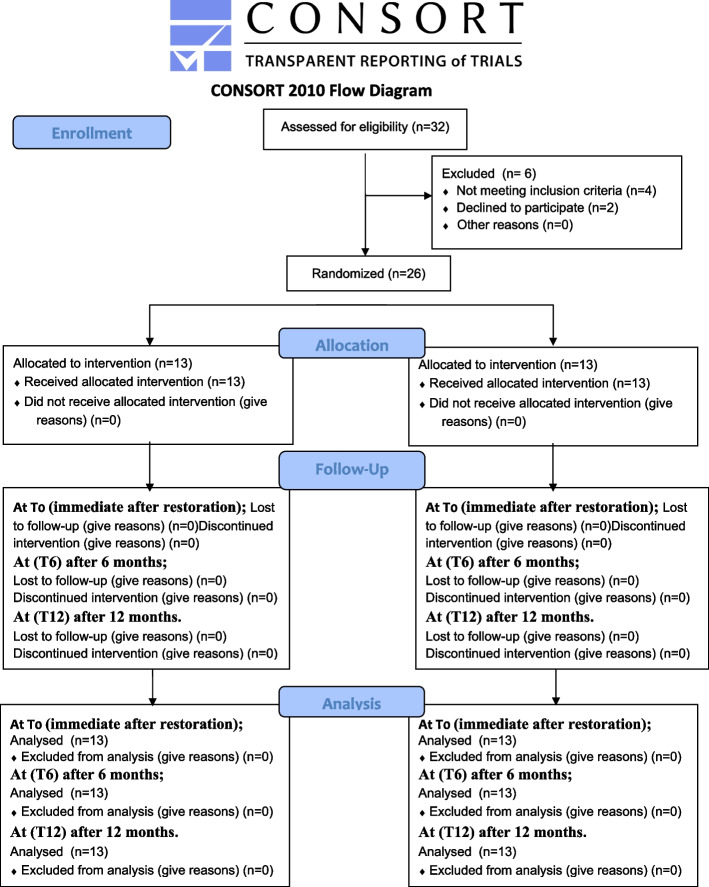
Consort flow diagram

This study is a randomized controlled clinical trial with two arms parallel design and having 1:1 allocation ratio, the study design follows the consort 2010 flow diagram. Figure 1.

### Eligibility criteria

The eligibility criteria of the enrolled participants were set as follows**:** For inclusion criteria of participants, participants eligible for the trial were males or females with age ranging from 35 – 50 years old and must comply with Cl I and Cl II loss of papillary height according to Nordland and Tarnow classification in maxillary teeth in esthetic zone, good oral hygiene, a good acceptable or controlled medical condition, cooperative participants who agreed to engage in this trial and signed the informed consent and participants that comply and assure to follow the clinical dental instruction. However, for Exclusion criteria of participants were set as the presence of uncontrolled parafunction habits, insufficient oral hygiene condition, presence of periodontal and gingival diseases, pregnant and lactating females as hormonal changes may have an impact on their periodontal condition, systemic diseases like Diabetes Mellitus or severe medical complications as they had effects on the periodontal condition, participants with mental and physical disabilities and participants undergo periodontal surgery to avoid any variations in the healing process of the papilla.

Inclusion criteria of teeth were Cl I and Cl II loss of papillary height according to Nordland and Tarnow classification in maxillary anterior teeth and vital upper anterior teeth with no signs or symptoms of irreversible pulpitis eliminate the chance of development of any endo-perio lesion that may act as a cofounder in this trial. Exclusion criteria of teeth were Cl III loss of papillary height according to Nordland and Tarnow classification in maxillary teeth in esthetic zone, Cl I,II,III loss of papillary height according to Nordland and Tarnow classification in mandibular teeth in esthetic zone, periapical pathosis or signs of pulpal pathology, multiple spacing, presence of orthodontic appliances or devices, presence of diastemas, non-vital tooth, periodontal affection associated with horizontal bone loss or tooth indicated for extraction.

### Sample size calculation

This power analysis used an esthetic anatomical form score as the primary outcome. The effect sizes (W) of the comparator (celluloid conventional matrix method) equal 1 and the effect size of the intervention (bio clear matrix) equals 0.8 were calculated based upon the results of Ergin et al., (2018) [11] and estimated proportions for the experimental group based upon expert opinion. Using alpha (α) level of (5%) and Beta (β) level of (20%) i.e. power = 80%; the minimum estimated sample size was a total of 21 subjects. The sample size was increased to 26 subjects (13 subjects per group) to compensate for a dropout rate of 25%. Sample size calculation was performed using G*Power Version 3.1.9.2**.**

### Recruitment

Eligible participants who fulfilled eligibility criteria were recruited from the outpatient clinic of the Conservative Dentistry department in the Faculty of Dentistry, Cairo University according to the participant timeline**.** The 26 participants were divided into two groups where: group (A) represents individuals with a black triangle that was restored with resin composite using a conventional celluloid matrix method, while group (B) received restorations with injection molding techniques using bioclear matrix.

### Allocation of participants

#### Random sequence generation (Randomization)

Randomization was assigned for eligible participants by generating numbers from 1:26 using Random Sequence Generator, Randomness and Integrity Services Ltd (http://www.random.org/). Each generated random number represented assigned to tooth intervention and comparator to each participant in a random manner.

#### Allocation concealment mechanism

The generated random numbers were placed in an opaque sealed envelope. The primary investigator carried out all the restoration procedures of this trial by picking one of these envelopes to avoid operator bias.

#### Blinding (masking)

Due to a discrepancy in the technique application protocol, the participants' assessors and statisticians were blinded to the techniques assignment, while the operator was not.

#### Participants’ selection

All participants were examined and selected based on the inclusion and exclusion criteria and informed consent was signed by all eligible patients participating in this clinical trial. The main complaints of the participants, as well as the teeth involved in this investigation, were diagnosed. The embrasure cervical to the interproximal contact that is not filled by gingival tissue is referred to as black triangles. For each participant mapping of the missed papilla was registered. All missed papillae in the esthetic zone (following inclusion criteria) were restored, and then a random selection of the restored missed papilla was included in the data collection (minimum one and maximum five).

#### Participants’ preparation

Intraoral photographs with the standard-setting of the Canon EOS 800D camera (exposure time 1\125 s, iso speed 100, and focal length 55 mm) and periapical radiographs were first taken for the selected participants. The treatment plan was then developed, and all participants received scaling and polishing using medium grit fluoride-free polishing paste (PSP prophy paste, UK) with Colorful Nylon Polishing Brush (Azdent, china) in a low-speed contra-angle hand-piece (E-type Contra Angle, NSK, Japan). An oral hygiene training program was provided to all participants before a treatment based on official ADA dental health recommendations (www.mouthhealthy.org).

#### Tooth preparation and restorative procedures

The materials used in this clinical trial, their descriptions, manufacture and lot number were mentioned in Table 1.

**Table 1:** List of materials, their description, lot number, and manufacturer website

**Materials**	**Description and composition**	**Lot no**	**Manufacture (website)**
**Select HV etch Bisco**	35% phosphoric acid gel	2,100,001,026	www.bisco.com
**Futura bond M + Voco**	Self-Etch Adhesive (Universal adhesive) acidic adhesive monomer (10–25% bisphenol A Glycidyle methacrylate), 2.5–5% organic acids, 10–25% cross-linking monomer (functionalized 2 hydroxyethyl methacrylate, catalyst (organic amine compound), initiator (camphorquinine), stabilizers, 10–25%solvent (ethanol and water)	2,049,202	www.voco.dental
**Grandio Voco**	Universal Nano-Hybrid resin composite, high glass filler content of 87% w/w, 2.5%-5%bisphenol A Glycidyle methacrylate, 2.5%-triethelene gycol dimethacrylate,2.5% Urethane dimethacrylate,	1,924,134	www.voco.dental
**Polofil NHT flow Voco**	Flowable light-curing nano-hybrid filling material, high filler content (> 76% w/w), 5–10% hydroxyethyldimethacrylate (HEDMA), 5–10% fumed silica, 2.5–5% bisphenol A Glycidyle methacrylate, 2.5%-triethelene gycol dimethacrylate		www.voco dental

#### Celluloid conventional matrix (Comparator group)

All procedural steps were performed by the primary investigator with the benefit of magnification (univet prismatic loupes 6.0 × 400 with AIRX with/red MKU60 with LED EOS 2.0 s). A standard shade guide (VOCO Grandio SO, Germany) was utilized to pick the appropriate shade of restorative material, and a blue 6*6 heavy rubber dam (Sanctuary Powder Free Latex Dental Dam, Malaysia) with universal canine & premolar clamp No.1 stainless steel (DENTECH KSK, Japan) was employed for isolation because adequate isolation is necessary for biofilm staining with the revealing agent and subsequent mechanical removal with an aluminum oxide spray.

#### Biofilm determination and removal

Bioclear dual-color disclosing solution was applied to the thoroughly dried teeth using a micro brush applicator (Cotisen, China) and was left for 30 s to set followed by rinsing; as the newer plaque stains were pink and the plaque older than 24 h were stained purple. Then blasting with (aluminum oxide air–water slurry) aluminum oxide powder Jeep sandblasting device was performed until the entire biofilm was removed.

#### Matrix insertion

The matrix strip** (**TOR VM, Russia) was inserted between the teeth deep into the gingival sulcus then, was checked for inciso-gingival height to assure proper building of the resin composite material involving all the emergence profile of the tooth.

#### Bonding procedure

**Fig. 2:**
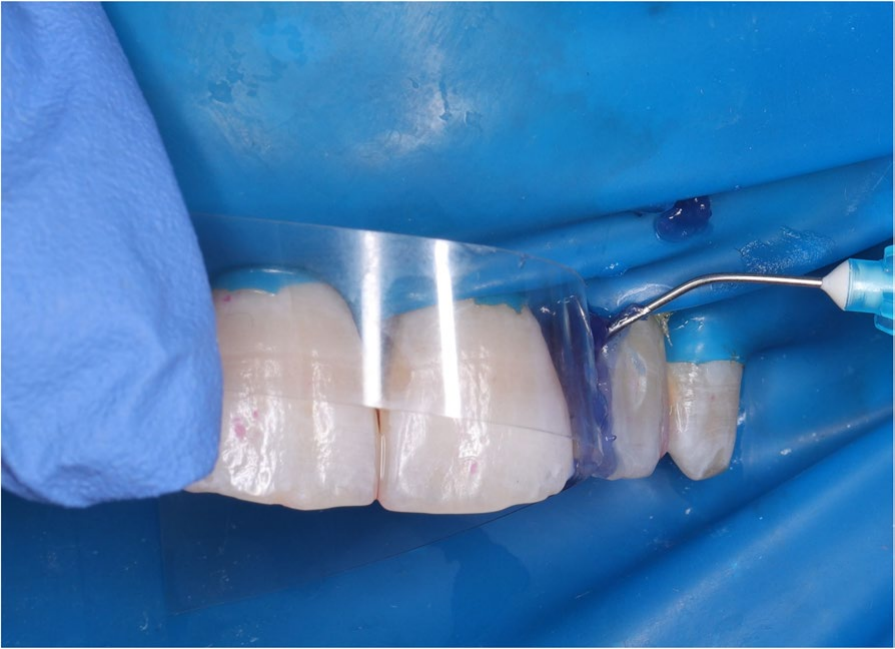
Enamel etching for conventional celluloid matrix group

The entire tooth was etched for 20 s with 35% phosphoric acid gel (Select HV etch, BISCO, USA), the acid etch agent was aspirated then rinsed with water for approximately 20 s, then excess moisture dried off with a gentle stream of air with triple way syringe until the chalky white appearance of the etched enamel.(Fig. 2) The bonding agent (FuturaBondM + , Voco, Germany) was applied by rubbing it with a bond disposable hard brush (Ivoclar Viva dent, Germany) for 20 s according to manufacture instructions then cured by a light-curing device (B-Cure Woodpecker Co., Ltd, Guilin Guangxi, China) that has an output of 1200 MW/cm^2^. The tip of the light-curing unit was held perpendicular to the tooth surface at zero distance to ensure optimal curing. The intensity of the curing light was monitored by a radiometer before application for each participant to guarantee that the light output was never less than 1200 Mw/cm^2^.

#### Resin composite application

Using a hand instrument (CompoRoller™, Kerr, Switzerland), the resin composite increments were carefully inserted between the matrix strip and the tooth. The matrix was then gently closed facially with slight incisal direction starting from the gingival aspect to ensure proper adaptation of the resin composite at the gingival area and to prevent gingival overhanging, Regular paste resin composite (Universal Nano-Hybrid resin composite Grandio Voco) was applied and light-cured. The restorations were gradually built up by layering composite under the strip to achieve the final shape and contour. The symmetric tooth was created similarly after the final contour of the proximal surfaces was produced.

#### Finishing the resin composite

Finally, the restorations were finished using superfine diamond polishing burs with yellow rings (EF504, Hanzhong Rising, Co., Ltd. Shaanxi, Hanzhong, China) followed by Sof-Lex abrasive discs (3 M Espe, 3 M/ESPE, St. Paul, MN, USA) were used to polish the resin composite build-ups and Kenda polishing kit (Kenda, Liechtenstein, Germany).

#### Post-operative instructions

The treatment was completed by instructing the participants how and where to clean their new restorations with a toothbrush and dental floss as part of their oral hygiene routine. Moreover, the participants were informed about the recall visits of follow-up periods, intraoral photographs were collected at the baseline and different follow-up periods to support further evaluation [12].

#### Bioclear matrix and injection molding (Intervention group)

All the upcoming procedural steps using bioclear matrix were performed according to manufacturer instructions. Optimum curvature for gingival embrasure closure was determined by a color-coded gauge and matrices that was corresponding to colors at the top of the matrix as following the gauge entered into the black triangle reveals the curvature to utilize. The matrices are available in two sizes (big and small incisors) and four curvatures, allowing the operator to treat the entire anterior sextant, canine to canine, and both upper and lower arches with robust and aesthetically acceptable results.

Black Triangle Matrix Series consists of color-coded matrices that used paired and closes space up to 2.5 mm, these series are available in two dimensions. Small used for mandibular incisors and small maxillary lateral incisors while Large ones are taller wrapping the tooth more than the small incisors matrices, large maxillary lateral incisors matrices, maxillary and mandibular canine matrices.

#### Gap sizing

**Fig. 3:**
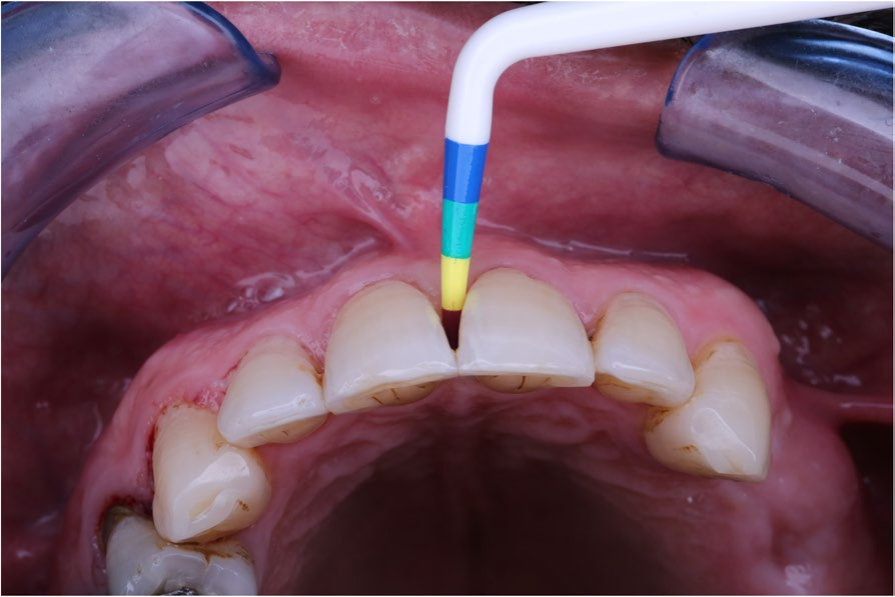
Gap sizing for bioclear matrix group

The black triangle Gauge was inserted under the contact into the black triangle space from facial to lingual until the gauge binds the incisal edges. These were a good reference to look from the occlusal for matrix color selection, in some cases, the gauge was in between colors in these cases using the smaller matrix in curvature (Fig. 3). After deciding if the tooth is small or large, the appropriate matrix in this size was selected.

#### Biofilm determination and removal

Bioclear dual-color disclosing solution was applied to the thoroughly dry teeth using a brush applicator letting it set followed by rinsing as the newer plaque stains were pink and the plaque older than 24 h and cracks were stained purple, then blasting with (aluminum oxide air–water slurry) aluminum oxide powder with Jeep sandblasting device until the entire biofilm was removed.

#### Contact optimization

True contact saw and sanders were used for contact cleaning and managing tension of contact as needed.

#### Matrix insertion

**Fig. 4:**
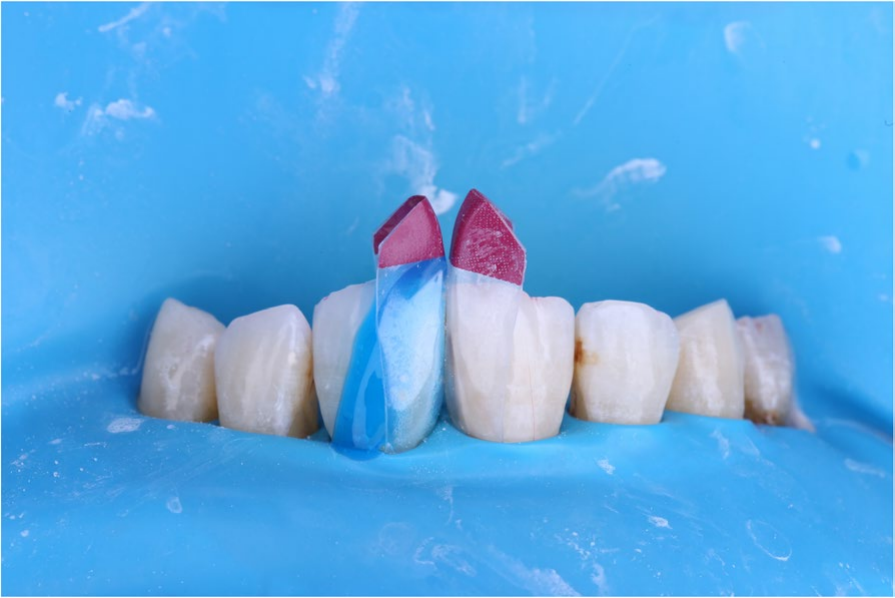
Enamel etching for bioclear matrix group

The determined appropriate pair matrices by the black triangle gauge were inserted. (Fig. 4).

#### Bonding procedure

The entire tooth was etched for 20 s with 35% phosphoric acid gel (Select HV etch, BISCO, USA). The acid etch agent was aspirated then rinsed with water for approximately 20 s, then excess moisture dried off with a gentle stream of air with triple way syringe until the chalky white appearance of the etched enamel. The bonding agent (FUTURABOND M + , VOCO, Germany) was applied by rubbing it with a bond disposable hard brush (Ivoclar Viva dent, Germany) for 20 s according to manufacture instructions.

#### Injection molding

Following the placement of the bonding agent, a small amount of heated flowable resin composite (Polofil NHT flow Voco, Germany) at 70 °C in dental resin composite heater (The Active Resin (AR) Heat, China) was carefully injected into both teeth to fill this critical cervical area to act as flowable resin composite reservoir, followed by the injection of heated Paste resin composite (universal Nano-Hybrid composite Grandio Voco, Germany). The snowplow technique or the injection moulding technique are the terms used to describe this procedure. A paste composite was then injected from the facial into the previously placed flowable composite reservoir, displacing as much of the flowable composite as possible into the palatal area.

Excess palatal material was removed prior to the curing device usage (B-Cure Woodpecker Co., Ltd, Guilin Guangxi, China). It was used to cure all three resin components (bonding resin, flowable composite, and paste composite) at the same time. The light-curing units’ tip was held perpendicular to the tooth surface at zero distance to ensure optimal curing. A radiometer was then used to measure the intensity of the curing light before and after it was applied to each participant, ensuring that the light output was never less than 1200 Mw/cm2. The marginal interface was sculpted and polished with utmost caution to avoid over-finishing.

#### Matrix releasing

The matrices were completely released from the teeth through grapping it by hemostate**.**

#### Finishing the resin composite

Finally, the restorations were finished following the same sequence as previously described in the conventional comparator group to blend the overmolded resin composite with the tooth surface.

#### Follow-up and assessment protocol

The restoration was assessed clinically by two experienced calibrated assessors immediately, after 6 months and one year. Calibration sessions were arranged for the two examiners prior to the initiation of the study as only experienced examiners with sufficient training and after proper calibration at ≥ 85% level will guarantee a reproducible result. Training procedures involves measuring the extent to which examiners record the same scores for the same phenomena before the beginning of study in order to reach kappa values of intra-examiner and inter-examiner agreement of ≥ 0.8. The primary, secondary, and tertiary outcomes were assessed following the FDI criteria as follows; esthetic evaluation as a primary outcome, marginal integrity as a secondary outcome, and patient satisfaction as a tertiary outcome. Esthetic evaluation involves the esthetic anatomical form, proximal anatomical form, periodontal response, and marginal staining. The secondary outcome involves clinical and radiographic marginal adaptation. The third outcome involves the patient's view in the forms of phonetics and food impaction [11, 12].

The outcomes of this clinical trial were assessed by the magnifying loupes in the form of a scoring system. For esthetic evaluation, the proximal anatomical form was given a score (1) when the contact point was normal and the floss could pass. Score (2) was given when the contact was slightly too strong, while the floss could pass. For the esthetic anatomical form, score (1) was given when the form was ideal according to width length ratio as the teeth length and width were measured by the digital caliper. Score (2) when there was slightly deviated from normal, and score (3) was given when the form deviates from normal but was still esthetically acceptable.

Regarding periodontal response (adjacent mucosa and oral and general health) it was assessed by the presence of plaque and gingival inflammation, plaque assessment was done by using the bioclear disclosing agent. Score (1) represent the absence of plaque and inflammation. Score (2) where there was little plaque without gingival inflammation. Score (3) was obtained when the plaque accumulation was at an acceptable level and gingival bleeding was acceptable the restoration took score (3). For marginal staining score (1) when there was no staining. Score (2) was obtained when there was minor staining but easily removable. While score (3) was obtained with moderate staining.

Radiographic marginal integrity and adaptation were assessed by using digital radiography, standardized radiographs were taken preoperative and immediately after the restoration (baseline), six months and one year later using an anterior parallel periapical kit. The Digora Optime DXR-50 001 digital intraoral imaging plate system (SoredexFinland) and Size 2 photostimulable phosphor plate (Soredex Corp., Tuusula, Finland) were used. The x-ray machine (Minray, Soredex-Finland) was programmed with the following settings: 70kVp, 7 mA, and 0.12 s exposure time. The exposure parameters were set for all patients at the start and throughout the study. The image plate was scanned by the Digora Optime DXR-50 001 (Soredex-Finland) digital scanner after the patient was radiographed. The Digora for Windows (DFW) 2.5 software program was used to analyze the images that were displayed on the computer monitor (Soredex-Finland).score (1) was obtained when no pathology harmonious transition between restoration and tooth, score (2.1) was given when the excess was acceptable, score (2.2) was representing positive\negative step at margin smaller than 150 μm, score (3.1) was obtained when the marginal gap was smaller than 200 μm, and score (3.2) was giving when negative steps visible smaller than 250 μm with non-noticable adverse effects, score (3.3) was representing a poor radio-opacity of the filling material. The score (4.1) was representing marginal gap larger than 250 μm, the score (4.2) was representing excess accessible but not removable, the score (4.3) was representing negative step larger than 250 μm but repairable, the score (5.1) was representing secondary caries or large gaps and the score (5.2) was representing the loss of restoration.

Marginal adaptation was assessed by visual-tactile examination with an FDI probe, score (1) was obtained when the restoration was with a harmonious outline, no gaps, and no discoloration, and (2.1) score was given when the marginal gap was (50 μm), (2.2) score was representing small marginal fracture removable by polishing, (3.1) score was obtained when the gap was smaller than 150 μm and not removable, 3.2 several small enamel or dentin fracture (4.1) was representing gap larger than 250 μm or dentin/based exposed, (4.2) score was representing chipping damaged margin, (5.1) score was given when the restoration was partial loose but in situ. (5.2) score was representing generalized major gap or irregularities. The tertiary outcome (patient view) in the forms of phonetics and food impaction, score (1) was obtained when the participant was entirely satisfied.

**Fig. 5:**
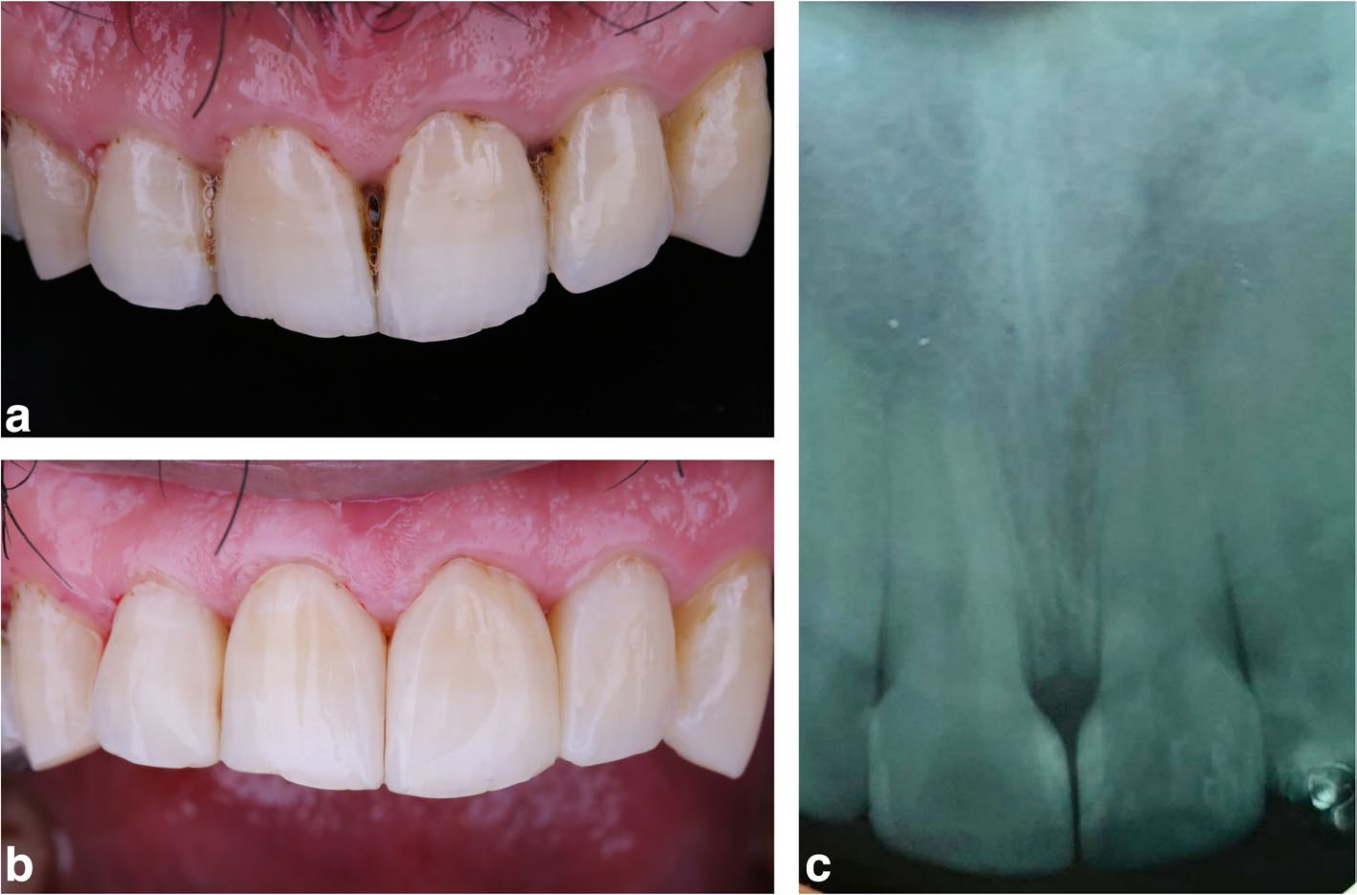
a epresentative preoperative photograph for black triangle between maxillary central incisors for bioclear matrix group. **b** representative postoperative photograph for black triangle between maxillary central incisors for bioclear matrix group after one year follow up period. **c** representative postoperative radiograph for black triangle between maxillary central incisors for bioclear matrix group after one year follow up

**Fig. 6:**
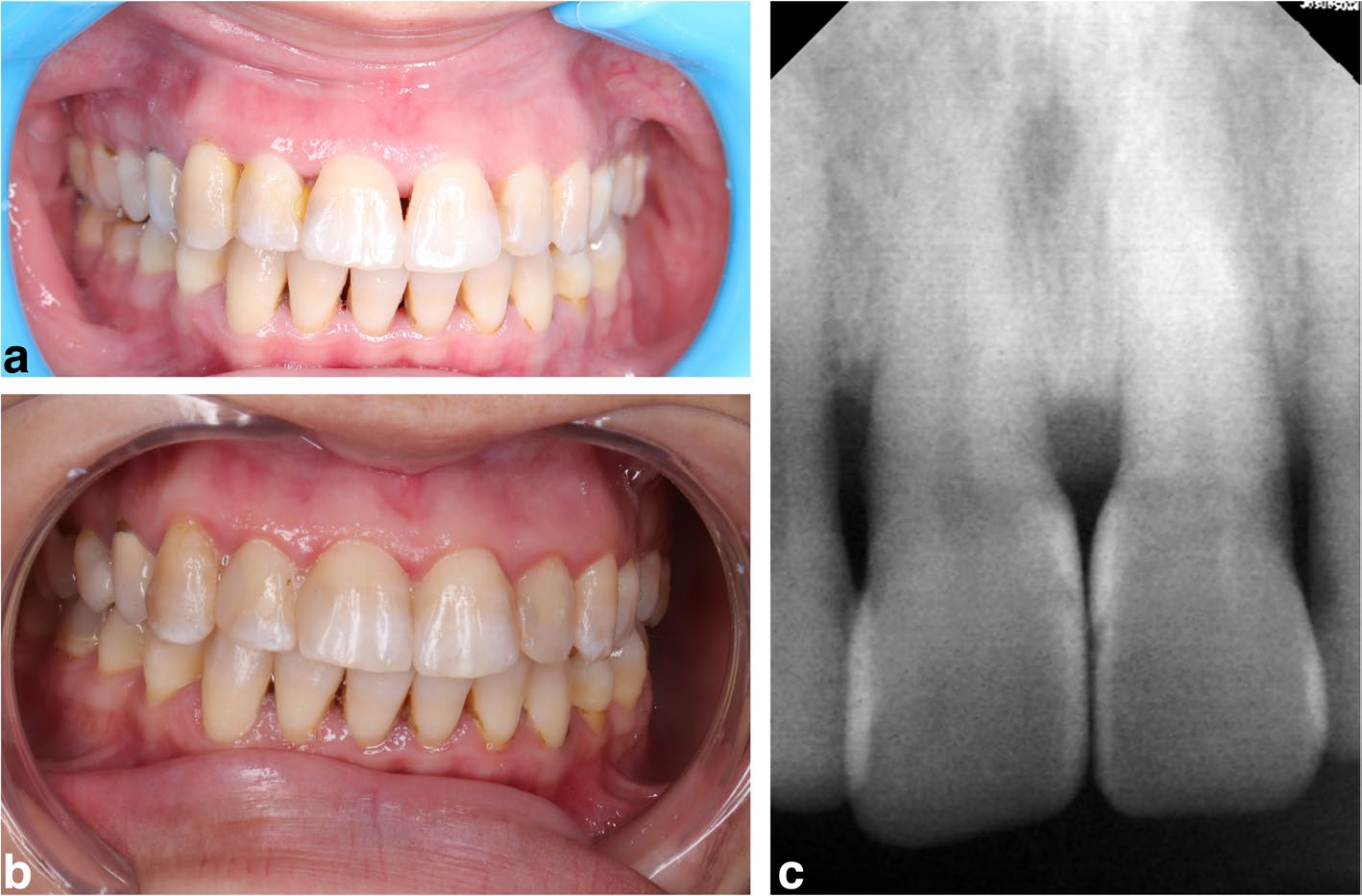
a Representative preoperative photograph for black triangle between maxillary central incisors for conventional celluloid matrix group. **b** representative postoperative photograph for black triangle between maxillary central incisors for conventional celluloid matrix group after one year follow up. **c** representative postoperative radiograph for black triangle between maxillary central incisors for conventional celluloid matrix group after one year follow up

The evaluation was done at baseline (T0) (immediate after restoration); (T6) after 6 months; (T12) after 12 months, each assessor at a time using a predesigned chart. Patients were instructed to brush their teeth regularly based on ADA dental health recommendations using a soft toothbrush (Oral-B Toothbrush Ultrathin, Oral-B, USA) and regular fluoridated toothpaste (Signal Cavity Fighter Toothpaste, USA) that were provided to the participants by the operator every six months on recall periods and follow-up assessment visits. Restorations were given scores of 1–5, where 1 is clinically excellent, and 5 is clinically poor. Restorations receiving scores 1–3 are considered successful, while 4 and 5 are failed restorations requiring repair or replacement (Fig. 5a,b,c) and (Fig. 6a,b,c).

### Statistical analysis

Age data were analyzed for normality using Shapiro–Wilk test and were found to be normally distributed so they were presented as mean and standard deviation values and were compared using independent t-test. Categorical and ordinal data were presented as frequency and percentage values. Categorical data were compared using fisher’s exact test. Intergroup comparisons for ordinal data were analyzed utilizing Mann–Whitney U test, while intragroup comparisons were analyzed using Friedman’s test followed by Nemenyi post hoc test. The significance level was set at p ≤ 0.05 within all tests. Statistical analysis was performed with R statistical analysis software version 4.1.3 for Windows [13].

## Results

### Demographic data

The study was conducted on 26 cases that were equally and randomly allocated to each of the studied groups (i.e. 13 cases each). There was 5(38.5%) males in the group (A) and 8(61.5%) females, while in group (B) there was 10(76.9%) males and 3(23.1%) females and the difference between groups was not statistically significant (*p* = 0.111). The mean age of the participants in group (A) was (41.77 ± 7.04) years, and in the group (B), it was (46.00 ± 9.93) years and there was no significant difference between both groups (*p* = 0.222). Demographic data were presented in Table 2.

**Table 2:** Demographic data

***Parameter***			***Group (A)***	***Group (B)***	***p-value***
***Sex***	***Male***	**n**	5	10	**0.111**
		**%**	38.5%	76.9%	
	***Female***	**n**	8	3	
		**%**	61.5%	23.1%	
***Age (years)***	***Mean*** ** ± ** ***SD***		41.77 ± 7.04	46.00 ± 9.93	**0.222**

### Esthetic evaluation

For different esthetic parameters, there was no significant difference between both groups in different follow-up intervals (*p* > 0.05). For proximal and esthetic anatomical forms, there was no significant difference between values measured at different intervals (*p* > 0.05), while for periodontal response and marginal staining the difference was statistically significant (*p* < 0.001). For periodontal response, post hoc pairwise comparisons for both groups showed value measured baseline to be significantly lower than values measured at different intervals (*p* < 0.001). For marginal staining in group (A) they showed value measured after 12 months to be significantly higher than baseline value (*p* < 0.001). While for group (B) they showed baseline value to be significantly lower than values of other intervals (*p* < 0.001). Esthetic evaluation parameters were presented in Table 3.

**Table 3:** Esthetic evaluation

***Interval***	***Score***	***Proximal anatomical form***				***p-value***	***Esthetic anatomical form***				***p-value***	***Periodontal response***				***p-value***	***Marginal staining***				***p-value***
		**Group (A)**		**Group (B)**			**Group (A)**		**Group (B)**			**Group (A)**		**Group (B)**			**Group (A)**		**Group (B)**		
		**n**	**%**	**n**	**%**		**n**	**%**	**n**	**%**		**n**	**%**	**n**	**%**		**n**	**%**	**n**	**%**	
***T0***	**1**	11	84.6%	13	100.0%	**0.166**	3	23.1%	4	30.8%	**0.208**	12^A^	92.3%	13^A^	100.0%	**0.356**	13^A^	100.0%	13^A^	100.0%	**1**
	**2**	1	7.7%	0	0.0%		4	30.8%	7	53.8%		0	0.0%	0	0.0%		0	0.0%	0	0.0%	
	**3**	1	7.7%	0	0.0%		6	46.2%	2	15.4%		1	7.7%	0	0.0%		0	0.0%	0	0.0%	
	**4**	0	0.0%	0	0.0%		0	0.0%	0	0.0%		0	0.0%	0	0.0%		0	0.0%	0	0.0%	
	**5**	0	0.0%	0	0.0%		0	0.0%	0	0.0%		0	0.0%	0	0.0%		0	0.0%	0	0.0%	
***T6***	**1**	11	84.6%	13	100.0%	**0.166**	3	23.1%	4	30.8%	**0.208**	1^B^	7.7%	0^B^	0.0%	**0.636**	4^AB^	30.8%	6^B^	46.2%	**0.192**
	**2**	1	7.7%	0	0.0%		4	30.8%	7	53.8%		6	46.2%	9	69.2%		6	46.2%	7	53.8%	
	**3**	1	7.7%	0	0.0%		6	46.2%	2	15.4%		6	46.2%	4	30.8%		3	23.1%	0	0.0%	
	**4**	0	0.0%	0	0.0%		0	0.0%	0	0.0%		0	0.0%	0	0.0%		0	0.0%	0	0.0%	
	**5**	0	0.0%	0	0.0%		0	0.0%	0	0.0%		0	0.0%	0	0.0%		0	0.0%	0	0.0%	
***T12***	**1**	11	84.6%	13	100.0%	**0.166**	3	23.1%	4	30.8%	**0.208**	1^B^	7.7%	0^B^	0.0%	**0.558**	3^B^	23.1%	5^B^	38.5%	**0.148**
	**2**	1	7.7%	0	0.0%		4	30.8%	7	53.8%		5	38.5%	4	30.8%		2	15.4%	4	30.8%	
	**3**	1	7.7%	0	0.0%		6	46.2%	2	15.4%		6	46.2%	9	69.2%		7	53.8%	4	30.8%	
	**4**	0	0.0%	0	0.0%		0	0.0%	0	0.0%		1	7.7%	0	0.0%		1	7.7%	0	0.0%	
	**5**	0	0.0%	0	0.0%		0	0.0%	0	0.0%		0	0.0%	0	0.0%		0	0.0%	0	0.0%	
***p-value***		**1**		**1**			**1**		**1**			< 0.001*		< 0.001*			< 0.001*		< 0.001*		

Values with different superscript letters within the same vertical column are significantly different *significant (*p* < 0.05)

### Marginal integrity

For both parameters in different follow-up intervals, group (A) had significantly higher score than group (B) (*p* < 0.05). While in both groups, there was no significant difference between values measured in different intervals (*p* > 0.05). Marginal integrity parameters were presented in Table 4.

**Table 4:** Marginal integrity

***Interval***	***Score***	***Radiographic marginal integrity***				***p-value***	***Marginal adaptation***				***p-value***
		**Group (A)**		**Group (B)**			**Group (A)**		**Group (B)**		
		**n**	**%**	**n**	**n**		**n**	**%**	**n**	**%**	
***T0***	**1**	2	15.4%	10	76.9%	0.004*	9	69.2%	13	100.0%	0.037*
	**2.1**	4	30.8%	1	7.7%		3	23.1%	0	0.0%	
	**2.2**	2	15.4%	1	7.7%		0	0.0%	0	0.0%	
	**3.1**	1	7.7%	0	0.0%		1	7.7%	0	0.0%	
	**3.2**	0	0.0%	1	7.7%		0	0.0%	0	0.0%	
	**3.3**	4	30.8%	0	0.0%		0	0.0%	0	0.0%	
	**4.1**	0	0.0%	0	0.0%		0	0.0%	0	0.0%	
	**4.2**	0	0.0%	0	0.0%		0	0.0%	0	0.0%	
	**4.3**	0	0.0%	0	0.0%		0	0.0%	0	0.0%	
	**5.1**	0	0.0%	0	0.0%		0	0.0%	0	0.0%	
	**5.2**	0	0.0%	0	0.0%		0	0.0%	0	0.0%	
***T6***	**1**	2	15.4%	9	69.2%	0.015*	8	61.5%	13	100.0%	0.017*
	**2.1**	4	30.8%	1	7.7%		3	23.1%	0	0.0%	
	**2.2**	2	15.4%	1	7.7%		1	7.7%	0	0.0%	
	**3.1**	1	7.7%	0	0.0%		1	7.7%	0	0.0%	
	**3.2**	0	0.0%	2	15.4%		0	0.0%	0	0.0%	
	**3.3**	4	30.8%	0	0.0%		0	0.0%	0	0.0%	
	**4.1**	0	0.0%	0	0.0%		0	0.0%	0	0.0%	
	**4.2**	0	0.0%	0	0.0%		0	0.0%	0	0.0%	
	**4.3**	0	0.0%	0	0.0%		0	0.0%	0	0.0%	
	**5.1**	0	0.0%	0	0.0%		0	0.0%	0	0.0%	
	**5.2**	0	0.0%	0	0.0%		0	0.0%	0	0.0%	
***T12***	**1**	2	15.4%	9	69.2%	0.026*	9	69.2%	13	100.0%	0.037*
	**2.1**	4	30.8%	1	7.7%		2	15.4%	0	0.0%	
	**2.2**	2	15.4%	1	7.7%		0	0.0%	0	0.0%	
	**3.1**	1	7.7%	0	0.0%		1	7.7%	0	0.0%	
	**3.2**	0	0.0%	1	7.7%		0	0.0%	0	0.0%	
	**3.3**	4	30.8%	0	0.0%		0	0.0%	0	0.0%	
	**4.1**	0	0.0%	0	0.0%		0	0.0%	0	0.0%	
	**4.2**	0	0.0%	0	0.0%		0	0.0%	0	0.0%	
	**4.3**	0	0.0%	0	0.0%		0	0.0%	0	0.0%	
	**5.1**	0	0.0%	0	0.0%		1	7.7%	0	0.0%	
	**5.2**	0	0.0%	1	7.7%		0	0.0%	0	0.0%	
***p-value***		**1**		**0.368**			**0.607**		**1**		

^*^significant (*p* < 0.05)

### Patient satisfaction

For both parameters in different follow-up intervals, both groups had a score of (1). Patient satisfaction parameters were presented in Table 5.

**Table 5:** Patient satisfaction

***Interval***	***Score***	***Phonetics***				***p-value***	***Food impaction***				***p-value***
		**Group (A)**		**Group (B)**			**Group (A)**		**Group (B)**		
		**n**	**%**	**n**	**%**		**n**	**%**	**n**	**%**	
***T0***	**1**	13	100.0%	13	100.0%	**1**	13	100.0%	13	100.0%	1
	**2**	0	0.0%	0	0.0%		0	0.0%	0	0.0%	
	**3**	0	0.0%	0	0.0%		0	0.0%	0	0.0%	
	**4**	0	0.0%	0	0.0%		0	0.0%	0	0.0%	
	**5**	0	0.0%	0	0.0%		0	0.0%	0	0.0%	
***T6***	**1**	13	100.0%	13	100.0%	**1**	13	100.0%	13	100.0%	**1**
	**2**	0	0.0%	0	0.0%		0	0.0%	0	0.0%	
	**3**	0	0.0%	0	0.0%		0	0.0%	0	0.0%	
	**4**	0	0.0%	0	0.0%		0	0.0%	0	0.0%	
	**5**	0	0.0%	0	0.0%		0	0.0%	0	0.0%	
***T12***	**1**	13	100.0%	13	100.0%	**1**	13	100.0%	13	100.0%	**1**
	**2**	0	0.0%	0	0.0%		0	0.0%	0	0.0%	
	**3**	0	0.0%	0	0.0%		0	0.0%	0	0.0%	
	**4**	0	0.0%	0	0.0%		0	0.0%	0	0.0%	
	**5**	0	0.0%	0	0.0%		0	0.0%	0	0.0%	
***p-value***		**1**		**1**			**1**		**1**		

### Clinical and radiographic outcomes

After 12 months, there is a single failed case in group (A) regarding periodontal response, marginal staining and adaptation. While in group (B) after the same interval, there was a single failed case regarding radiographic marginal integrity. For other parameters, all cases in different intervals were successful. Clinical and radiographic outcomes were presented in Table 6.

**Table 6:** Clinical and radiographic outcomes

**Parameter**	***Interval***	***Failed cases***				***Risk ratio (95%CI)***	***Odds ratio (95%CI)***	***p-value***
		**Group (A)**		**Group (B)**				
		**n**	**%**	**n**	**%**			
***Proximal anatomical form***	**T0**	0	0.0%	0	0.0%	**NA**	**NA**	**NA**
	**T6**	0	0.0%	0	0.0%	**NA**	**NA**	**NA**
	**T12**	0	0.0%	0	0.0%	**NA**	**NA**	**NA**
***Esthetic anatomical form***	**T0**	0	0.0%	0	0.0%	**NA**	**NA**	**NA**
	**T6**	0	0.0%	0	0.0%	**NA**	**NA**	**NA**
	**T12**	0	0.0%	0	0.0%	**NA**	**NA**	**NA**
***Periodontal response***	**T0**	0	0.0%	0	0.0%	**NA**	**NA**	**NA**
	**T6**	0	0.0%	0	0.0%	**NA**	**NA**	**NA**
	**T12**	1	7.7%	0	0.0%	**0.33 (0.01:7.5)**	**0.31 (0.01:8.3)**	**1**
***Marginal staining***	**T0**	0	0.0%	0	0.0%	**NA**	**NA**	**NA**
	**T6**	0	0.0%	0	0.0%	**NA**	**NA**	**NA**
	**T12**	1	7.7%	0	0.0%	**0.33 (0.01:7.5)**	**0.31 (0.01:8.3)**	**1**
***Radiographic marginal integrity***	**T0**	0	0.0%	0	0.0%	**NA**	**NA**	**NA**
	**T6**	0	0.0%	0	0.0%	**NA**	**NA**	**NA**
	**T12**	0	0.0%	1	7.7%	**3 (0.13:67.52)**	**3.24(0.12:87.13)**	**1**
***Marginal adaptation***	**T0**	0	0.0%	0	0.0%	**NA**	**NA**	**NA**
	**T6**	0	0.0%	0	0.0%	**NA**	**NA**	**NA**
	**T12**	1	7.7%	0	0.0%	**0.33 (0.01:7.5)**	**0.31 (0.01:8.3)**	**1**
***Phonetics***	**T0**	0	0.0%	0	0.0%	**NA**	**NA**	**NA**
	**T6**	0	0.0%	0	0.0%	**NA**	**NA**	**NA**
	**T12**	0	0.0%	0	0.0%	**NA**	**NA**	**NA**
***Food impaction***	**T0**	0	0.0%	0	0.0%	**NA**	**NA**	**NA**
	**T6**	0	0.0%	0	0.0%	**NA**	**NA**	**NA**
	**T12**	0	0.0%	0	0.0%	**NA**	**NA**	**NA**

*NA* Not Applicable

## Discussion

The first and most basic goal of restorative dentistry is to preserve the tooth structure. However, for long-term restoration survival, the periodontium should be healthy, or vice versa [14]. Periodontal tissues are critical to the proper aesthetics, function, and convenience of dentition. As an initial requirement for clinical success, the appearance of a healthy periodontium is critical to all conservative, endodontic, and prosthetic therapies. Periodontology and restorative dentistry interact in several clinical areas, including the interface between the position of the restoration margins, the contours of the dental crown, and the subsequent response of the gingival tissues to conservative and prosthetic restorations [15].

Restorative management of the black triangle represents almost 67% of the adult population over the age of 20 years [2]. There were limited numbers of articles related to this restorative management that were restricted to case series and case reports, hence, this clinical trial would add to the adoption or rejection of these management protocols.

In the current trial, teeth were assigned into intervention or control groups using simple randomization 1: 1 with the help of computer software (https://www.random.org/). The baseline data regarding age, gender, and tooth type, did not affect outcomes because both groups were equally randomized and there were no substantial differences found among these different variables.

As ascertained by Schulz, (2001) [16], the researchers used allocation concealment to avoid selection bias by preventing them from affecting which participants were assigned to the intervention or control groups. It was done by numbering opaque sealed envelope.The current study was a blinded study, where participants and the assessor were blinded. As cited by Higgings et al., (2015) [17], blinding of participants and personnel reduces performance bias. The assessors who were not involved in sequence generation, allocation concealment, or treatment options were the blinded assessors.

This step ensures standards of quality, improves performance, and provides credibility of the study.. Clinical trials must go through a rigorous process before being accepted. The investigation was carried out with the acceptance of the institutional review board of the Faculty of Dentistry, Cairo University, as it is recommended that before a clinical trial can take place, a group of researchers who are not participating in the trial should first approve the protocol, the overall plan, and all phases of the trial. This is called an independent scientific review or peer review.

As hybrid resin composites were confirmed to have better overall performance in terms of esthetics and fracture than microfilled resin composites [11]. In this regard, nanohybrid resin composite (Grandio, Voco) was utilized in the current study. Resin composite materials have the advantage of being available several stable shades with great wear-resistant [18]. A case management of black triangle was presented by Clark in order to restore phonetics and enhance the esthetic appearance by minimizing the open gingival space by restorative regenerative papilla (RRP approach. In this approach build up the emergence profile of the tooth using flowable composite in conjunction with Bioclear matrix instead of resin composite paste material thus reducing the interproximal space between teeth as itwould be nearly impossible to place a paste material in such a claustrophobic area without voids and without disturbing the anatomically shaped matrices [4, 7].

Kim and Clark, (2017) [8] claimed that treatment of black triangle using bioclear method was a predictive additive non-invasive simple technique, and could be easily learned by all clinicians. This technique provide smooth contour of the restoration subgingivally, adequate surface finish surface and minimal calculus accumulation thus greater protection of the root from repeated scaling and reducing root sensitivity. In addition to orthodontic stability was achieved by branding the proximal contact in both direction incisogingivally and buccolingually.,Because black triangle disease is a growing concern for patients, they suggested that the available treatment options are unpredictable and can be biologically costly. However, the bioclear method enables a clinician to treat black triangles by focusing on white instead of pink*,* and macro-aesthetics of a smile. This non-invasive method attains patients’ desires, with the advantages of simplicity and predictable results as it can be learned and applied by the average clinicians [8].

The use of a clear mylar strip also known as celluloid matrix is the most popular matrix technique for proximo-incisal defects as the mylar strip technique originated with the beginning history of the resin restorations, where bulk material had to be placed because of the short working time of chemically cured resin materials. While for light curing materials, provided the clinicians extended controlled working time allowing for incremental insertion technique. Hence the Celluloid matrix is simple, available, cheap, and there is no need for special equipment considered to be their main advantages [5].

Restoring the correct emergence profile of the teeth with adequate proximal contact is considered as on the the greatest challenges for clinicians. Thus, several studies assessed different materials and techniques in order to properly establishing the tightness and quality of the proximal contact, and balancing the teeth as well as, the health of the periodontium [6].

In this clinical trial, in order to obtain a standardized process it was very important to standardize the several variables as patient selection by following the inclusion criteria and patient preparation even an air abrasion step was done for both groups to exclude this variable.

In order to minimize cofounders and eliminate selection bias black triangles were selected in maxillary teeth in the esthetic zone due to increased variability in form, contour, and arrangement of teeth between upper and lower arch to reflect restorative procedure and interdental papillary regeneration., Cl III loss of papillary height according to Nordland and Tarnow classification in maxillary teeth in esthetic zone was excluded because this method is unpredictable when a large volume of tissue is missing, this was in accordance to Parihar, (2018) [19].

All the restorative procedures were carried with magnification loops as magnification establishes a squared, instead of a linear, relationship between magnification powers and "picture elements" or "pixels" of information. In other words, clinicians operating at 3.5 × see 10 times more visual information than with the naked eye, and clinicians working at 10 × see 100 times more [7]**.**

Rubber dam isolation allow for retraction of soft-tissue and papilla compression. Furthermore it protects the soft tissues during sandblasting process to remove sticky biofilm. For lateral compression to existing papillae, a minimum subgingival restorative contour of 1.0- to 1.5-mm is required. This lateral papillary compression mold the papilla to fill the embrasure space not filled by resin composite [20–22]. Biofilm removal and determination were done following Swanson, (2018) [23]. When bonding to large enamel areas, total-etch remains the most robust method, especially on uncut enamel [24].

In this clinical trial, in order to restore the proximal profile as a restorative approach for management of the black triangle two techniques were established and compared, the conventional celluloid matrix technique and bioclear matrix system. According to the conventional celluloid matrix technique, the matrix strip** (**TOR VM, Russia) was inserted between the teeth deep into the gingival sulcus and was checked for inciso-gingival height to assure proper building of the resin composite material involving all the emergence profile of the tooth no wedge was used as the usage of wedges create a flat cervical shape which lacks the static pressure needed for papillary regeneration and the spatula tip of the comporoller was used in order to contour the increments to ensure adequate gingival embrasure and emergence profile following Vargas, (2010) [21].

As the celluloid matrix is flaccid, its manipulation was not easy. Care was taken not to pull the strip too tightly, to prevent under contouring of restoration. It was initially over-contoured in order to facilitate finishing to an ideal contour. The narrowed Mylar strip was useful for controlling emergence and gingival contour. Also, it was easy to access resin instruments and improved visibility following Azzaldeen et al., (2014) [25].

Intervention group using bioclear matrix was performed according to manufacturer instructions to guarantee the standardization of the procedure. Before the application of the rubber dam application, a critical step was performed where the contacts were sanded in order to lighten the tension at the areas of contacts and allow full seating of the matrices. Moreover, contacts sanding allow for removal of the soft and sticky biofilm present around the contact area that is not removed by etching process as well as the fluoride pellicle on the tooth for ideal bonding performance. Flowable and paste resin composites were heated to 70 c^o^ for ideal flow. Wedge placement is neither recommended nor necessary as suggested by Kim and Clark, (2017) [8].

The inactive matrix used on the adjacent teeth “Shield matrix” was used to protect and maintain the embrasure shape as well as keep the adjacent tooth from resin contamination during injection molding. The Shield matrix was used after a while as active one [8].

The anatomical contour of the matrix allows for a smooth cervical curvature, leading to proper direct resin composite designs that are highly conductive to papilla regeneration. This is due to two inherent characteristics. The first feature is the ability to use the papilla like a wedging force instead of a traditional wedge. A traditional wedge shapes the cervical into a flat shape. Flat cervical shapes lack the static pressure required for papilla regeneration. The second distinguishing feature is an anatomically correct shape with exaggerated palatal, interproximal, and facial surfaces. This allows the clinician to simply remove the matrix following photo-polymerization, with minimum or even no interproximal finishing. Tissue health can be excellent even with a very round embrasure form when the finish is extremely smooth and there is no gingival ledge. This modern perspective on cervical curvature contrasts sharply with the outdated belief that prosthetic and restorative embrasures should be flat [9]. Resin composite finishing was done by a new kit for every single patient for standardization.

FDI criteria were used in this trial to clinically evaluate the different outcomes of the study, as FDI criteria were described as being practical, relevant, and standardized, making comparisons between parameters easier. Investigators should continue to use them to improve the standardization of their clinical judgments and to allow comparisons with other studies. Photographs were provided to both examiners as reference tools to illustrate scoring at each criterion [26].

This was a randomized clinical trial, which is typically regarded as the paradigm for clinical trials and provides the highest level of evidence. As cited by Hopewell et al. (2010) [27], randomization minimizes selection bias and confounders that was revealed by the demographic data analysis where the participants in the two groups were similar as possible in terms of general characteristics such as age, and gender, with the major difference remaining between them being their exposure to different protocols.

In the present study, the null hypothesis was accepted for the outcomes: proximal anatomical form, Esthetic anatomical form, periodontal response, and marginal staining, phonetics and food impaction. However it was rejected for the outcomes: radiographic marginal integrity and marginal adaptation. The results of the clinical evaluation of proximal anatomical form and food impaction revealed that all cases in both groups were successful, with no statistically significant difference. In the present study, group A (celluloid matrix) showed successful aesthetic performance. This might be attributed to proper handling of the matrix and steady flow of the materials as the celluloid matrix was inserted between the teeth down into the gingival sulcus and checked for inciso-gingival length to ensure proper resin composite material construction involving the entire emergence profile of the tooth. The findings were consistent with the previous investigations of Ergin et al., (2018) [11] and Kim, (2019) [10]. Furthermore, group B (bioclear matrix) showed successful scores, which were attributed to the proper selection of the bioclear matrix size according to the bioclear gauge and complete seating of the matrix after using the true contact [10].

In terms of the evaluation of aesthetic anatomical form, the difference found between celluloid matrix and bioclear matrix was not statistically significant. In the present study, the majority of cases in group A (celluloid matrix) reported a score of (3) at all intervals, denoting that form deviates from normal but is aesthetically accepted, while most of the cases in group B (bioclear matrix) had a score of (2), denoting form only slightly deviates from the normal. According to Bansal et al., (2019) [28] and Korkut and Unal, (2021) [29], proper finishing, polishing, and proper selection of resin composite type in the form of nanohybrid resin composite were associated with successful restorative management. This justifies the success of all cases in both groups. Throughout all follow-up intervals, the difference in aesthetic anatomical form between the celluloid matrix and bioclear matrix groups was not statistically significant. The bioclear matrix group, on the other hand, had the greatest aesthetic anatomical form scores.

Regarding the periodontal response, at T0 (immediately after restoration), the majority of the cases in the celluloid matrix group and all the cases in bioclear matrix group reported a score of (1), denoting no plaque and no inflammation. At T6 (6 months), the majority of the cases in the celluloid matrix group either reported a score of (2), denoting little plaque and no inflammation, or a score of (3), denoting plaque accumulation or acceptable gingival bleeding, while most of the cases in bioclear matrix group had scored (2). At T12 (12 months), the majority of the cases in both groups reported a score of (3). These results were in accordance with Kim and Clark. (2017) [8] who restored the black triangle with bioclear matrix and obtained a favorable tissue response. At T0 ( immediately after restoration) and T6 (6 months), all cases in both groups were successful, while at T12 (12 months), there was a single failed case in the celluloid matrix group as it was clinically unsatisfactory but reparable.

At all-time intervals, the difference in periodontal response between the celluloid matrix and bioclear matrix groups was not statistically significant. However, there was a statistically significant difference between scores measured at different follow-up intervals. At T0 (immediately after restoration), it was significantly different from other intervals. This might be attributed to placing the restorations' margins slightly below the gingival crest to achieve a natural appearance, precise application of composite resin material, resin composite excess removal, polishing of all resin composite surfaces may have reduced their impact on adjacent oral tissues, the teeth were isolated using a rubber dam to allow for better visualization and tissue retraction, which would aid in relating the restoration contour to the proximal tissues in a more periodontium-friendly approach; and post-operative instruction to the participants to follow oral hygiene measures strictly with the prohibition of using toothpicks.

Regarding the marginal staining, at T_0_ (immediately after restoration), all cases in both groups reported a score of (1), denoting no staining. At T_6_ (6 months), the majority of the cases in both groups reported a score of (2), denoting minor staining and easily removable. At T_12_ (12 months), the majority of the cases in celluloid matrix group reported a score of (3), denoting moderate staining that may also present on other teeth but not aesthetically unacceptable, and most of the cases in bioclear matrix group had scored (1).

At all-time intervals, the difference in marginal staining between the celluloid matrix and bioclear matrix groups was not statistically significant. This was attributed to the absence of bone loss, no overhang or gap formation, good periodontal condition, no signs and symptoms, no cervical caries, no roughness, and immediate flash removal. This finding has been attributed to the fact that two groups received the same resin composite and finishing and polishing protocol with the same operator.this result was following Mattar et al., (2016) [30].

However, there was a statistically significant difference between scores measured at different follow-up intervals. In celluloid matrix group, at T_0_ (immediately after restoration), it was significantly different from the T_12_ (12 months) interval. In group B (bioclear matrix), at T_0_ (immediately after restoration), it was significantly different from different intervals. At T_0_ (immediately after restoration) and T_6_ (6 months), all cases in both groups were successful, while at T_12_ (12 months), there was a single failed case in the celluloid matrix group as it was clinically unsatisfactory but reparable.

In terms of the marginal adaptation, there was a statistically significant difference between both groups’ at all follow-up intervals. However, there was no statistically significant difference between the scores measured at different follow-up intervals. The majority of the cases in group A (celluloid matrix) and all the cases in group B (bioclear matrix) reported a score of (1), denoting harmonious outline, no gaps, and no discoloration was found. At T_0_ (immediately after restoration) and T_6_ (6 months), all cases in both groups were successful, while at T_12_ (12 months), there was a single failed case in the celluloid matrix group.

Regarding the radiographic marginal integrity results, there was a statistically significant difference between both groups’ at all follow-up intervals. However, there was no statistically significant difference between the scores measured at different follow-up intervals for both groups. These results came in agreement with Clark and Kim. (2017) [8] and Kim. (2019) [10] who got smooth subgingival restorative contours showed in radiographs after using bioclear matrix.

The majority of the cases in group A (celluloid matrix) reported either a score of (2.1), denoting acceptable excess present, or (3.3), denoting poor radio-opacity of the filling material, and all the cases in group B (bioclear matrix) reported a score of (1), denoting no pathology harmonious transition between restoration and tooth. This was attributed to the flaccid nature of the celluloid matrix, unlike the appropriate anatomic shape with exaggerated palatal, interproximal and facial surfaces and the self-stabilizing design of the bioclear matrix. The Heated flowable and paste resin composite material fills the mold in the form of bioclear matrix perfectly with a nice transition to the tooth structure due to its viscosity. It can thus be molded in a very thin layer, eliminating the need for tooth preparation in most cases and preserving the entire healthy tooth structure, as in the presented case. To be more specific, because it is a completely additive technique, only the etch-and-rinse protocol is required for effective adhesion [31].In addition, no caries was observed adjacent to the restoration margins in any of the two tested groups. At T_0_ (immediately after restoration) and T_6_ (6 months), all cases in both groups were successful, while at T_12_ (12 months), there was a single failed case in the bioclear matrix group.

As the science of phonetics implies how sounds and speech are articulated, gingival tissue should cover the teeth's roots, but if exposure occurs, particularly interproximal, speech may be affected as air goes through the missed papilla space [32]. In both groups, all cases had a score of (1), denoting entirely satisfied patients at all follow-up intervals, which were considered successful. Adding to this, there was a positive effect and no problem associated with sound pronunciation. The findings were consistent with previous literature by Shankar, (2012) [18] and Chhavia and Sandeep, (2017) [33].

Within the limitations of the present research, it could be considered that the restorative management of the black triangle with both protocols meets the following objectives: superior aesthetic and good marginal adaptation; suitable biological properties; enhanced subjective and objective appearance of the treated teeth; and adequate survival time. Thus the null hypothesis was accepted for the outcomes: proximal anatomical form, Esthetic anatomical form, periodontal response, and marginal staining, phonetics and food impaction. However it was rejected for the outcomes: radiographic marginal integrity and marginal adaptation. Both techniques are clinically relevant and were almost equally successful, however they are depending on the operator skills. Thus some clinical recommendation could be addressed as guide for clinicians:

1. For celluloid matrix technique, to achieve the ideal emergence profile, contact point, and correct contour and to prevent overhanging, ensure cervical strip closure in the apical direction.
2. For bioclear matrix technique, respect the size of the matrix (color) in accordance with the gauge for an optimum result.

### Further investigations:

1. Further investigations are required to assess the clinical use of celluloid mylar strip and bioclear matrix.
2. Further trials are required in mandibular teeth cases.
3. Further investigations concerning an interdisciplinary approach for severe cases should be taken into consideration.
4. Since the FDI criteria customized scale is considerably more applicable for evaluating the clinical outcomes, additional research utilizing this scale is necessary.
5. Further studies are required to assess gingival tissue volume of interdental papilla.

## Conclusions

Within the limitations of this study, it can be concluded that:

Both restorative protocols had efficient management of the black triangle in terms of participant esthetics satisfaction, positively overcame food impaction, anatomically correct tissue contours were possible and affected phonetics properly at different follow up periods.

A highly finished and polished resin composite aids in regeneration of missed interdental papillae while causing no harm to them.

The bioclear matrix provided an appropriate marginal adaptation. Additionally, over molding techniques maintain the usage of oral hygiene measures by blending the resin composite margin to the tooth surface to create accessible margins. As for Celluloid matrix is available, cheap, and there is no need for special equipment. On the other hand, it is a sensitive technique and needs high skills.

Prognostic factors influencing successful restoration outcomes include not only the technique of restoration but also periodontal maintenance and follow-up.

## Data Availability

The data that support the findings of this study are available from the corresponding author, on reasonable request.

## Abbreviations

FDI: Fédération Dentaire International (International Dental Federation)
HA: Hyaluronic Acid
IDP: Inter Dental Papilla
MSC: Mesenchymal Stromal Cells
PCA: Proximal Contact Area
PCAP: Proximal Contact Area Proportion
PRP: Platelet- Rich Plasma
SEI: Smile Esthetic Index
